# A General Electrode Design Strategy for Flexible Fiber Micro‐Pseudocapacitors Combining Ultrahigh Energy and Power Delivery

**DOI:** 10.1002/advs.201700003

**Published:** 2017-03-03

**Authors:** Ping Li, Jing Li, Zhe Zhao, Zhengsong Fang, Meijia Yang, Zhongke Yuan, You Zhang, Qiang Zhang, Wei Hong, Xudong Chen, Dingshan Yu

**Affiliations:** ^1^ Key Laboratory for Polymeric Composite and Functional Materials of Ministry of Education, Key Laboratory of High Performance Polymer‐based Composites of Guangdong Province School of Chemistry Sun Yat‐sen University Guangzhou 510275 China; ^2^ Beijing Key Laboratory of Green Chemical Reaction Engineering and Technology Department of Chemical Engineering Tsinghua University Beijing 100084 China

**Keywords:** fiber electrodes, flexible energy storage, micro‐pseudocapacitors, MnO_2_, textile electrodes

## Abstract

Herein, a general strategy is proposed to boost the energy storage capability of pseudocapacitive materials (i.e., MnO_2_) to their theoretical limits in unconventional 1D fiber configuration by rationally designing bicontinuous porous Ni skeleton@metal wire “sheath–core” metallic scaffold as a versatile host. As a proof of concept, the 1D metallic scaffold supported‐MnO_2_ fiber electrode is demonstrated. The proposed “sheath” design not only affords large electrode surface area with ordered macropores for large electrolyte‐ion accessibility and high electroactive material loading, but also renders interconnected porous metallic skeleton for efficient electronic and ionic transport, while the metallic “core” functions as an extra current collector to promote long‐distance electron transport and electron collection. Benefiting from all these merits, the optimized fiber electrode yields unprecedented specific areal capacitance of 1303.6 mF cm^−2^ (1278 F g^−1^ based on MnO_2_, approaching the theoretical value of 1370 F g^−1^) in liquid KOH and 847.22 mF cm^−2^ in polyvinyl alcohol (PVA)/KOH gel electrolyte, 2–350 times of previously reported fiber electrodes. The solid‐state fiber micro‐pseudocapacitors simultaneously achieve remarkable areal energy and power densities of 18.83 µWh cm^−2^ and 16.33 mW cm^−2^, greatly exceeding the existing symmetric fiber supercapacitors, together with long cycle life and high rate capability.

The rapid advances in portable/wearable electronics have stimulated ever‐increasing demand in flexible miniature power modules.^1^, ^2^, ^3^ Solid‐state flexible fiber micro‐supercapacitors (m‐SCs) have gained a surge of attentions.^1^, ^2^, ^3^, ^4^, ^5^, ^6^ As for practical applications with microscale fiber devices, the areal performance is becoming an increasingly important evaluation metric, since the active material mass and the device volume are usually negligible.^7^ However, the major bottleneck for the existing fiber m‐SCs lies in their much lower areal energy density relative to routine planar SCs^8^ or batteries.^2^ In this context, considerable efforts were concentrated on exploring proper fiber electrode materials with large capacitance for improving the energy density, while maintaining high power density.^1^, ^4^, ^5^, ^6^, ^7^, ^8^, ^9^, ^10^, ^11^, ^12^, ^13^, ^14^, ^15^, ^16^, ^17^ Various carbonaceous materials like activated carbon,^9^, ^10^ carbon nanotubes (CNTs),^11^, ^12^, ^13^ reduced graphene oxide (rGO),^14^, ^15^, ^16^ and our recently developed rGO/CNT hybrids^17^, ^18^ were exploited as active materials for fiber m‐SCs, yet their applications are restricted by the low capacitance of <200 mF cm^−2^. Alternatively, incorporating pseudocapacitive materials into fiber m‐SCs is a superior solution to achieve high‐density energy due to 10–100 times higher theoretical capacitance than carbon materials.^19^, ^20^, ^21^, ^22^, ^23^, ^24^, ^25^, ^26^, ^27^, ^28^, ^29^, ^30^, ^31^, ^32^, ^33^, ^34^, ^35^, ^36^ Due to the poor conductivity for pseudocapacitive materials, composite electrode design by depositing active materials on one‐dimensional (1D) conductive scaffolds including carbon‐based fibers^19^, ^20^, ^21^, ^22^, ^23^, ^24^, ^25^, ^26^, ^27^, ^28^, ^29^, ^30^ or metal‐based wires,^31^, ^32^, ^33^, ^34^, ^35^, ^36^ was employed to improve the electron transport. Despite some progresses, the improvements in the areal energy density for these fiber m‐SCs are still too modest to cater for many practical requirements and often come at the expense of sacrificing their rate capability or power density.^1^, ^19^, ^20^, ^21^, ^22^, ^23^, ^24^, ^25^, ^26^, ^27^, ^28^, ^29^, ^30^, ^31^, ^32^, ^33^, ^34^, ^35^, ^36^ This impedes their broad practical applications where both high power and energy densities are required. Thus, further boosting the energy density of fiber micro‐pseudocapacitors while retaining high power delivery becomes an urgent yet challenging mission.

Relative to some macroscopic pseudocapacitors (>500 mF cm^−2^ and >10 mW cm^−2^),^37^, ^38^ the existing fiber micro‐pseudocapacitors demonstrated much inferior performance. In this regard, one main reason lies in that those previous 1D electrode scaffolds are not well‐engineered specifically aiming at unique fiber devices, thus the full demonstration of high theoretical pseudocapacitance of active materials is severely limited in unconventional 1D format, which becomes a hot yet unresolved issue recently. Actually, the 1D electrode scaffolds available for fiber pseudocapacitors are very limited. The widely employed scaffolds in the literature are mainly carbonaceous fibers such as CNT yarns, rGO fibers, and their composite fibers. But their conductivity is unsatisfactory for long‐range electron transport along the fiber.^19^, ^20^, ^21^, ^22^, ^23^, ^24^, ^25^, ^26^, ^27^, ^28^, ^29^, ^30^ Unlike macroscopic planar SCs, effective long‐distance electron transport is highly required for fiber m‐SCs due to the lack of extra metal current collectors within slender fiber electrodes. However, such crucial design concern is often ignored in previous carbon‐based fiber m‐SCs, thus leading to a major bottleneck in performance (typically <200 mF cm^−2^ and <1 mW cm^−2^).^19^, ^20^, ^21^, ^22^, ^23^, ^24^, ^25^, ^26^, ^27^, ^28^, ^29^, ^30^ As compared, metallic scaffold was recognized as the best candidate for long‐range electron transport.^31^, ^32^, ^33^, ^34^, ^35^, ^36^ For instances, bare Ni wires were often utilized as conductive skeletons to load various active materials like Ni(OH)_2_/rGO,[[qv: 36b]] CuCo_2_O_4_ nanowires,[[qv: 36c]] and rGO^15^ for fiber m‐SCs. However, these bare metallic wires are solid and often suffer from limited surface area,^31^, ^32^, ^33^, ^34^, ^35^, ^36^ also commonly seen in carbon‐based fiber electrodes.^19^, ^20^, ^21^, ^22^, ^23^, ^24^, ^25^, ^26^, ^27^, ^28^, ^29^, ^30^ This undoubtedly restricted allowable active material loading, since thicker active material layer may cause poor adhesion, inferior mechanical stability, and high resistivity. In principle, ideal 1D pseudocapactive fiber electrode should consist of high‐surface‐area, conductive and porous framework and concurrently render: (1) efficient long‐range electronic transport; (2) fast ion transport/diffusion; (3) a large fraction of active materials.^1^, ^2^ However, as mentioned above, those existing fiber electrodes using 1D metallic wires/or carbonaceous fibers failed to simultaneously satisfy the above three key requirements, thereby resulting in limited areal energy densities and inferior power output.^19^, ^20^, ^21^, ^22^, ^23^, ^24^, ^25^, ^26^, ^27^, ^28^, ^29^, ^30^, ^31^, ^32^, ^33^, ^34^, ^35^, ^36^ Thus, in order to develop advanced fiber pseudocapacitors combining high power and energy densities, it becomes particularly paramount to simultaneously fabricate and engineer 1D porous electrode scaffolds to maximize the potential of pseudocapacitive materials. However, this is a challenging mission to integrate high‐surface‐area, conductive pore network into tiny fiber electrodes due to constraints of fiber geometrical structures and flexibility requirements. To our knowledge, 1D porous fiber scaffolds enabling simultaneous optimization of both electron/ion transport and active material loading have not appeared yet in previous studies.

Herein, for the first time, we have designed and fabricated a new family of 1D hierarchically structured sheath–core metallic scaffold for micro‐pseudocapacitor applications. Such unique scaffolds are achieved by conformally assembling bicontinuous porous Ni framework as “sheath” onto various metal (Ni, Cu, stainless steel) wires as “core” via template‐directed assembly and electrodeposition. As a proof of concept, the hybrid fiber electrodes consisting of 1D “sheath–core” metallic scaffold and nanostructured MnO_2_ were demonstrated. The proposed unique electrode design not only affords the large electrode surface area with ordered macropores for large electrolyte‐ion accessibility and high electroactive material loading (>1 mg cm^−2^), but also renders interconnected porous metallic skeleton for rapid and efficient electronic and ionic transport for the electroactive materials. Significantly, the metallic Ni “core” can not only function as an extra current collector to promote long‐distance electron transport and electron collection efficiency, but also ensure the mechanical stability and flexibility of the whole fiber electrode. All the above merits enable the full utilization of the large theoretical capacitance for pseudocapacitive materials (e.g., MnO_2_) in fiber devices even at high charge/discharge rates and bending states. As anticipated, the newly produced prototype fiber electrodes with well‐engineered metallic scaffolds yielded a remarkable specific areal capacitance up to 1303.61 mF cm^−2^ in KOH liquid electrolytes at 1.69 mA cm^−2^. More importantly, the assembled solid‐state symmetric fiber micro‐pseudocapacitor with polyvinyl alcohol (PVA)/KOH gel electrolytes simultaneously achieved large specific areal capacitance of 847.22 mF cm^−2^, ultrahigh device energy and power densities of 18.83 µWh cm^−2^ and 16.33 mW cm^−2^ (75.32 µWh cm^−2^ and 65.32 mW cm^−2^ based on single electrode), greatly surpassing almost all previous solid‐state symmetric fiber SCs (typically 2.4–411 mF cm^−2^, 0.17–9.2 µWh cm^−2^, and 0.1–4.2 mW cm^−2^, Table S1, Supporting Information) and even some macroscopic planar pseudocapacitors,^1^, ^4^, ^5^, ^6^, ^7^, ^8^, ^9^, ^10^, ^11^, ^12^, ^13^, ^14^, ^15^, ^16^, ^17^, ^18^, ^19^, ^20^, ^21^, ^22^, ^23^, ^24^, ^25^, ^26^, ^27^, ^28^, ^29^, ^30^, ^31^, ^32^, ^33^, ^34^, ^35^, ^36^ together with very long cycle life as well as good rate capability and device flexibility. In principle, the resultant 1D metallic scaffold can be utilized as a flexible, highly conductive, and durable current collectors to host a range of active materials toward a maximum utilization for advanced fiber micro‐pseudocapacitors.

The fabrication procedure of the proposed 1D “sheath–core” electrode scaffold was schematically illustrated in **Figure** **1** (see also Supporting Information for the details). Typically, the opal template produced from 2 µm polystyrene spheres was first assembled onto the curved Ni wire (the diameter: 0.1 mm) surface by electrophoretic deposition (**Figure** **2**a,b). After sintering the template, nickel was electrodeposited throughout the polystyrene opal template. A nickel inverse opal framework as “sheath” consisting of numerous narrow interconnects between spherical voids was conformally formed on the solid Ni wire “core” after removing the template, as clearly observed in Figure 2c, which was realized by immersing the plated Ni wire into chloroform followed by the oxygen plasma treatment. Such bicontinuous architecture with ordered macroporosity from the porous Ni “sheath” is advantageous to optimize the active material loading in the fiber electrode, while the presence of the solid Ni “core” not only renders additional highly conductive “trunk” pathways for efficient long‐distance electron transport and improved electron collection, but also relieves the conflict between good mechanical flexibility and all porous frameworks. To our knowledge, this is the first report on the creation of highly conductive, interconnected pore framework within 1D fiber architecture. Finally, to exploit the merit of the proposed 1D electrode scaffold, manganese oxide—the most promising pseudocapacitive material—was electrodeposited into the porous nickel framework. It should be pointed out that anodic pulse deposition was adopted in the present work with an aim to guarantee a conformal coating of the active materials through such complex architecture and enable desirable mechanical and electrical contact of the plating active materials with the Ni backbone for facile electronic transport. As shown in Figure 2d–f, scanning electron microscopy (SEM) observation for the plated metal wire reveals interconnected porous metallic skeletons consisting of a thin conformal coating of nanostructured metal oxides (denoted as MnO_2_/porous Ni wire). Such unique microarchitecture design favors fast electron transport with shortened solid‐state ion diffusion path.^2^ The cross‐sectional SEM image indicates that the porous Ni “sheath” possesses a thickness of around 100 µm (Figure 2g,h). The energy dispersive X‐ray (EDX) element mapping analysis for both the outer surface and the cross‐section throughout the entire thickness exhibits a uniform dispersion of Mn element within such a complex architecture together with O and Ni elements (Figure 2i–l and Figure S1 (Supporting Information)), which verifying the conformal electrodeposition of MnO_2_ throughout the whole porous Ni skeleton. It is worth noting that interconnected porous Ni framework is still well maintained after the deposition of MnO_2_ (e.g., 1.0 mg cm^−2^) with desirable thickness, guaranteeing continuous ion transport pathways in the fiber electrode. X‐ray diffraction pattern of the plated metal wire suggests the poor crystallization and approximately amorphous characteristic of the as‐plated oxide owing to peak broadening and lack of sharp peaks (Figure S2, Supporting Information). X‐ray photoelectron spectroscopy result (Figure S3, Supporting Information) for the plated metal wire reveal the binding energies of Mn 2p_3/2_ and Mn 2p_1/2_ located at 654.0 and 642.2 eV, respectively, in good agreement with the literature results,^10^ indicative of the formation of manganese oxides, while the Mn 3s spectrum (Figure S3, Supporting Information) exhibits a peak separation value of 4.83 eV, indicating that Mn^4+^ is the main Mn species in the plated manganese oxides.^10^

**Figure 1:**
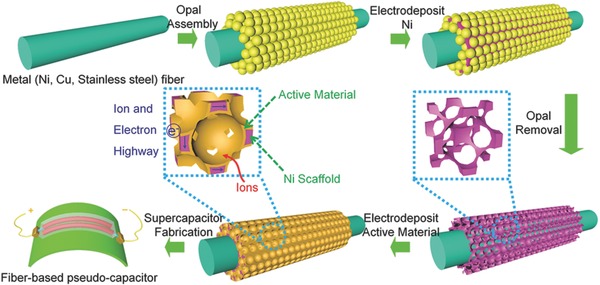
Schematic illustration for the fabrication of bicontinuous porous Ni framework sheathed 1D metallic scaffold supported‐MnO_2_ hybrid fiber electrode and the corresponding solid‐state fiber micro‐pseudocapacitor.

**Figure 2:**
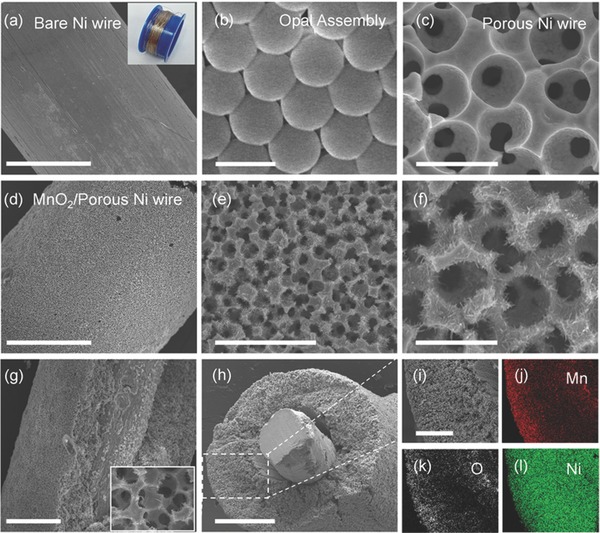
a) SEM image of the bare Ni wire. The inset is digital photograph of commercial Ni wires with a diameter of 0.1 mm. b) SEM image of assembled opal template from 2 µm polystyrene spheres on a bare Ni wire. c) SEM image of bicontinuous porous Ni framework sheathed metal Ni wire. d–f) SEM images of porous Ni framework sheathed metal Ni wire with a thin MnO_2_ plating layer. g,h) SEM images of the fractured surface and side‐section of the porous Ni framework sheathed metal Ni wire with a thin plating layer of MnO_2_. i–l) EDX mapping data of the square area highlighted in (h). Scale bars: 150 µm (a), 2.2 µm (b), 3 µm (c) and (f), 130 µm (d), 10 µm (e), 90 µm (g), 100 µm (h), 40 µm (i).

As anticipated, our 1D metallic scaffold electrodes with high electrical conductivity, large ion‐accessible surface area, and interconnected pore network are beneficial for high‐performance micro‐pseudocapacitors. The electrochemical properties of the MnO_2_/porous Ni wire electrode were first evaluated using a three‐electrode configuration in KOH aqueous solution (**Figure** **3**a). Clearly, all the cyclic voltammetry (CV) curves as a function of scan rate from 5 to 100 mV s^−1^ exhibit a roughly rectangular shape along with a couple of approximately symmetrical peaks at 0.11 and 0.17 V, respectively, similar to the literature results, which could arise from the intercalation/deintercalation of K^+^ in the MnO_2_ electrode in aqueous KOH.^39^, ^40^ Furthermore, the galvanostatic charge/discharge (GCD) curve of the MnO_2_/porous Ni wire electrode at various current densities (Figure 3b) shows a triangular‐like shape, indicating good capacitive behavior of the electrode. Various mass loading of MnO_2_ in the fiber electrode was investigated by varying the plating time ranging from 6 to 23 min. As can be seen in Figure S4a (Supporting Information), the areal mass loading of MnO_2_ was linearly increased from 0.32 to 1.29 mg cm^−2^, which is much higher those of previous 1D scaffolds such as Cu‐wire supported CuO@AuPd nanowhiskers.^35^ The length capacitance versus the plating time of MnO_2_ is plotted in Figure S4b (Supporting Information). At a scan rate of 5 mV s^−1^, increasing the plating time leads to the increase of the MnO_2_ loading, and therefore the length capacitance is enhanced until a saturation value is reached. Nevertheless, further increase in the plating time to 19 min results in the reduced capacitance. This indicates that the elongating plating time may engender the thicker MnO_2_ layer (Figure S5, Supporting Information). But the excessive MnO_2_ will prohibit the effective contact of MnO_2_ with the electrolyte and impedes the electron/ion transport. As a result, the best capacitive performance is achieved for a 17 min plating time, corresponding to a mass loading of 1.0 mg cm^−2^. For comparison, the bare porous Ni wire electrode without MnO_2_ was also investigated by the CV measurements (Figure S6, Supporting Information). The CV curve for the MnO_2_ plating electrode exhibits substantially higher current than that of the bare electrode (Figure S6, Supporting Information), illustrating the overwhelming contribution of the active MnO_2_, though bare Ni scaffold itself has a very small capacitance (vide infra).

**Figure 3:**
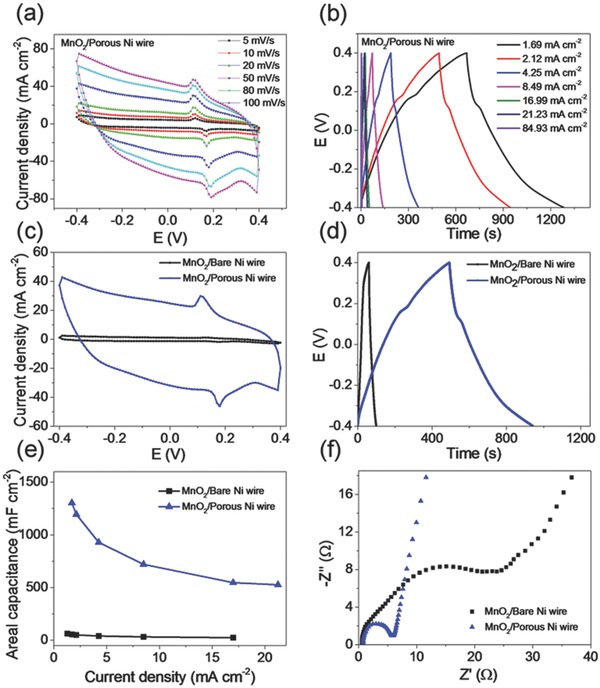
a) CV curves of the MnO_2_/porous Ni wire electrode at various scan rates in KOH. b) GCD curves of the MnO_2_/porous Ni wire at various current densities from 1.69 to 84.93 mA cm^−2^. c) Comparative CV curves of the MnO_2_/bare Ni wire electrode and the MnO_2_/porous Ni wire electrode measured at 50 mV s^−1^. d) Comparative GCD curves of MnO_2_/bare Ni wire electrode and MnO_2_/porous Ni wire electrode measured at 2.12 mA cm^−2^. e) Areal capacitance of the MnO_2_/bare Ni wire and MnO_2_/porous Ni wire electrode with increasing current densities. f) Nyquist plots of the MnO_2_/bare Ni wire electrode and the MnO_2_/porous Ni wire electrode with a frequency loop from 0.01 Hz to 100 kHz.

To illustrate the advantages of bicontinuous porous framework sheathed Ni wire electrodes, we perform a comparison study with bare Ni wire at the identical condition with the same MnO_2_ loading of 1.0 mg cm^−2^. The comparative CV results of the MnO_2_/bare Ni wire and the MnO_2_/porous Ni wire at 50 mV s^−1^ in KOH aqueous electrolyte are included in Figure 3c. Clearly, the MnO_2_/porous Ni wire electrode presents more than one order of magnitude larger current density than that of the MnO_2_/bare Ni wire electrode, verifying substantially improved capacitive performance for the newly designed electrode architectures. This is coincident with the comparative results from the GCD curves collected at the same current density (Figure 3d), which presents much longer discharge time for the MnO_2_/porous Ni electrode. Figure 3e compares the areal capacitance of the MnO_2_/bare Ni and MnO_2_/porous Ni electrodes. Remarkably, the MnO_2_/porous Ni wire electrode achieves a very high specific capacitance up to 1303.61 mF cm^−2^ at a current density of 1.69 mA cm^−2^, which is 21 and 51 times higher than those of the MnO_2_/bare Ni (61.94 mF cm^−2^) and the bare porous Ni electrode without MnO_2_ (25.50 mF cm^−2^) at the identical current density. This result highlights the particular advantage of the hierarchically structured metallic scaffold in advanced 1D hybrid electrodes, in which the porous Ni backbone renders rich metal/MnO_2_ interface with improved electronic transport of MnO_2_, and the interconnected open pore network guarantees sufficient contact between the electrolyte and the MnO_2_ as well as rapid ion transport.

To evaluate the contribution of MnO_2_ to the electrochemical performance of the MnO_2_/porous Ni wire electrode, the specific capacitance of MnO_2_ was calculated based on the subtraction of the capacitance belonging to the bare porous Ni skeleton from the total capacitance. The highest specific capacitance of ≈1278 F g^−1^ was achieved for a 17 min deposition of MnO_2_, approaching the theoretical value of MnO_2_ (≈1370 F g^−1^). This implies the ultrahigh utilization efficiency of the active MnO_2_ material in our well‐engineered 1D hybrid electrode. The Nyquist plot for the MnO_2_/porous Ni wire electrode as shown in Figure 3f exhibits a straight line nearly vertical to the real axis in the low frequency region, suggesting ideal capacitive behavior of the MnO_2_/porous Ni electrode. The equivalent series resistance for the MnO_2_/porous Ni electrode is as low as 0.5 Ω along with a much smaller semicircle in high frequency region implying a much lower charge‐transfer resistance and more facile ion diffusion compared to the MnO_2_/bare Ni wire electrode.

To further assess the performance of the MnO_2_/porous Ni wire hybrid electrode in practical energy storage devices, we used the optimized MnO_2_/porous Ni electrode to create flexible symmetrical micro‐SCs. Typically, two parallel fiber electrodes were mounted onto a polyester (PET) substrate with PVA/KOH as the electrolyte. As expected, the CV curves for the constructed fiber SC at various scanning rates (5–100 mV s^−1^) present a nearly rectangular shape in the potential window of 0–0.8 V (Figure S7, Supporting Information), while the GCD curves as a function of the applied current densities (**Figure** **4**a) have a triangular‐like shape with a small IR‐drop voltage, suggesting good reversibility and charge propagation in the fiber‐like format. Despite the fact that the solid‐state electrolytes have poorer ion diffusivity relative to the liquid electrolyte, the MnO_2_/porous Ni wire electrode still yields a remarkable specific areal capacitance as high as 847.22 mF cm^−2^ at 0.41 mA cm^−2^ with the corresponding length capacitance of 79.81 mF cm^−1^, which is substantially higher than those of previous MnO_2_‐based fiber electrodes such as MnO_2_/rGO fibers (9.6 mF cm^−2^),^24^ MnO_2_/CNT yarn (61.25 mF cm^−2^),^21^ polypyrrole(PPy)/MnO_2_/rGO/metal yarns (411 mF cm^−2^ at 0.092 mA cm^−2^),^31^ and other representative high‐performance fiber electrodes measured at much lower current densities (0.01–0.1 mA cm^−2^) such as hollow rGO/poly(3,4‐ethylenedioxythiophene):poly(styrenesulfonate)(PEDOT:PSS) fibers (304.5 mF cm^−2^ at 0.08 mA cm^−2^),^30^ CNT/Co_3_O_4_ fibers (52.6 mF cm^−2^ at 0.053 mA cm^−2^),^23^ CNT/polyaniline fibers (38 mF cm^−2^ at 0.01 mA cm^−2^),^27^ CNT/activated carbon yarns (31.7 mF cm^−2^ at 0.05 mA cm^−2^),^13^ and rGO/CNT@ carboxymethyl cellulose fibers (177 mF cm^−2^ at 0.1 mA cm^−2^),^5^ and even superior to some MnO_2_‐based macroscopic SCs using MnO_2_/nickel wire arrays/Ti foil (750 mF cm^−2^) and MnO_2_/nickel foam (557 mF cm^−2^).^41^, ^42^ More significantly, our fiber device can operate even at a very high current density up to 16.33 mA cm^−2^. The specific areal capacitance at 8.17 mA cm^−2^ can reach ≈50% capacitance retention of the value at 0.41 mA cm^−2^. Such high operation rates have not appeared yet in previous solid‐state fiber pseudocapacitors.^1^, ^19^, ^20^, ^21^, ^22^, ^23^, ^24^, ^25^, ^26^, ^27^, ^28^, ^29^, ^30^, ^31^, ^32^, ^33^, ^34^, ^35^, ^36^ The good rate performance could arise from the following reason: (1) the ordered macroporous structure feature enhances the infiltration of gel electrolytes and thus the ion diffusion; (2) the good mechanical and electrical contact between thin‐layer MnO_2_ nanostructures and porous conductive metallic “sheath,” which reduces interface impedance and facilitates fast electronic/ionic transport.^2^

**Figure 4:**
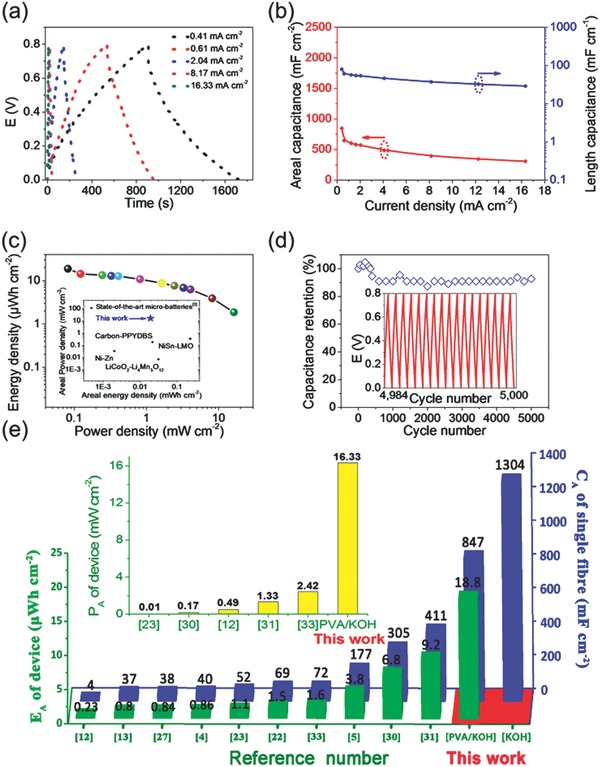
a) GCD curves of the solid‐state fiber SC based on MnO_2_/porous Ni wire at various current densities. b) The areal and length capacitances of the solid‐state fiber SC using MnO_2_/porous Ni wire from discharging profiles. c) Ragone plots based on the solid‐state fiber SC. The inset is the comparative results on the areal energy and power densities of our optimized fiber SC with state‐of‐the‐art microbatteries. d) Cycle life of the solid‐state fiber SC using MnO_2_/porous Ni wire. The inset is the GCD curves after 4984 cycles at 12.74 mA cm^−2^. e) Comparison of the electrochemical performances of our optimized fiber device with previous fiber‐shaped SCs in areal specific capacitance (*C*_A_), areal energy density (*E*_A_), and areal power density (*P*_A_).

Figure 4c presents a Ragone plot of the energy and power densities of our solid‐state fiber m‐SC based on the MnO_2_/porous Ni wire. As can be seen, our fiber device can deliver an impressively high areal energy density of 75.32 µWh cm^−2^ (based on single fiber electrode) and 18.83 µWh cm^−2^ (based on the entire device). Remarkably, the energy output value competes favorably with state‐of‐the‐art microbatteries based on Ni–Zn (2 µWh cm^−2^), carbon–polypyrrole‐dodecylbenzene sulphonic acid(PPYDBS) (21.45 µWh cm^−2^), and LiCoO_2_–Li_4_Mn_5_O_12_ (30.6 µWh cm^−2^),^2^ while the maximum power density is as high as 65.32 mW cm^−2^ (based on single electrode) and 16.33 mW cm^−2^ (based on the entire device), which is one to three orders of magnitude higher than those of microbatteries. Cycling stability is another important criterion for evaluating device performance. As can be seen from Figure 4d, our fiber device shows significant capacitance retention of 92.73% after 5000 charging–discharging cycles, demonstrating the superior cycling stability.

Figure 4e further compares the areal performance of our fiber pseudocapacitors and other fiber‐based SCs in the literature. The specific areal capacitance of our solid‐state fiber device with PVA/KOH electrolyte at 0.41 mA cm^−2^ is ≈2–350 times of previously reported solid‐state fiber SCs measured at much lower rates (typically 0.01–0.1 mA cm^−2^) reported so far (see Table S1, Supporting Information).^1^, ^4^, ^5^, ^6^, ^7^, ^8^, ^9^, ^10^, ^11^, ^12^, ^13^, ^14^, ^15^, ^16^, ^17^, ^18^, ^19^, ^20^, ^21^, ^22^, ^23^, ^24^, ^25^, ^26^, ^27^, ^28^, ^29^, ^30^, ^31^, ^32^, ^33^, ^34^, ^35^, ^36^ More importantly, both the areal energy and power densities of 18.83 µWh cm^−2^ and 16.33 mW cm^−2^ based on the device (75.32 µWh cm^−2^ and 65.32 mW cm^−2^ based on one electrode) are substantially higher than those of previous advanced fiber devices using hollow rGO/PEDOT: PSS fiber (6.8 µWh cm^−2^, 0.166 mW cm^−2^),^30^ rGO/MnO_2_/PPy@metal yarn (9.2 µWh cm^−2^, 1.5 mW cm^−2^),^31^ MnO_2_/CNT fiber (8.5 µWh cm^−2^),^20^ PPy@CNTs@urethane elastic fibers (6.13 µWh cm^−2^, 0.133 mW cm^−2^),^26^ GO/CNT@carboxymethyl cellulose fibers (3.84 µWh cm^−2^, 0.19 mW cm^−2^),^5^ and nanoporous Au wire@MnO_2_//CNT/carbon fibers (5.4 µWh cm^−2^, 2.53 mW cm^−2^).^36^ To our knowledge, the areal energy and power densities for our prototype device represent the highest values among all solid‐state symmetric fiber SCs (Table S1, Supporting Information). 1, 4, 5, 6, 7, 8, 9, 10, 11, 12, 13, 14, 15, 16, 17, 18, 19, 20, 21, 22, 23, 24, 25, 26, 27, 28, 29, 30, 31, 32, 33, 34, 35, 36

The fiber SC was further subjected to mechanical bending measurements at different bending states. It was found that the bending has negligible effect on the device capacitance on bending to 180° (**Figure** **5**a and Figure S8 (Supporting Information)), demonstrating good device flexibility desirable for flexible electronics. To meet specific energy/power requirements for various flexible device applications, three fiber SCs can be integrated on a PET substrate in series, exhibiting a 2.4 V voltage window relative to the single fiber SC that operated at 0.8 V (Figure 5b). What is more, three SCs connected in series can successfully power an electronic watch display (Figure 5c), illustrating great potential of our fiber device as efficient energy storage units. Not limited to metal Ni wires, our feasible method can be applicable to construct bicontinuous porous framework on other conductive substrates like Cu and stainless steel wires (Figures S9 and S10, Supporting Information). More significantly, our versatile strategy can also produce textile electrodes consisting of numerous 1D “sheath–core” fibers based on metal gauzes for energy storage textiles (Figure 5d and Figure S11 (Supporting Information)), which will greatly broaden their potential applications in wearable/portable energy storage systems.

**Figure 5:**
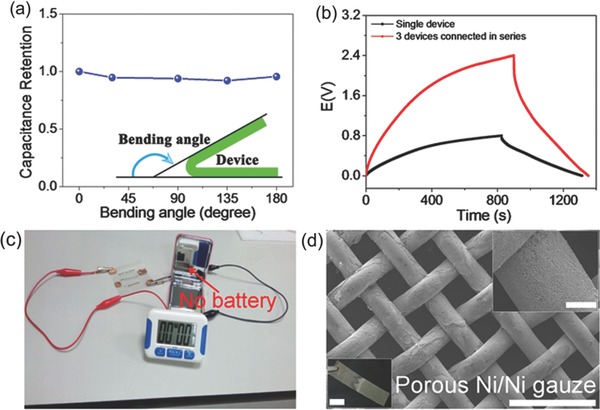
a) Capacitance retention of the solid‐state fiber SC using MnO_2_/porous Ni wire under different bending states. b) GCD curves of single fiber device and three fiber devices connected in series. c) Digital photograph of three fiber devices connected in series to power an electronic watch display. d) SEM images with low (scale bar: 500 µm) and high (scale bar: 50 µm) magnifications for a large‐area (1 cm × 5 cm) Ni gauze sheathed with porous Ni framework. The inset at the bottom left of (d) is the photograph of large‐area Ni gauze with the plating porous Ni (scale bar: 1 cm).

Clearly, the present work illustrated how a well‐engineered electrode scaffold significantly boosts the energy storage performance of pseudocapacitive materials in 1D fiber format. Unlike previous 1D carbonaceous/metallic scaffolds with a lack of the comprehensive design consideration for unique fiber devices, our electrode architecture largely satisfies the aforementioned essential requirements for a high‐performance fiber pseudocapacitor by affording high active material loading and high utilization efficiency for high energy density and simultaneously rendering efficient electronic and ionic transport as well as short solid‐state diffusion length for high‐power output. This judicious design can also cater to a range of pseudocapacitive materials including conductive polymers and metal oxides and represents a general and appealing strategy to make a full utilization of high theoretical pseudocapacitance of active materials and achieve a trade‐off between energy and power densities that leads to advanced microscale power sources. Furthermore, there is still plenty of room to further optimize the electrochemical performance of the prototype device by varying electroactive materials, tuning the “sheath” thickness and pore size parameters, yielding fiber devices with even higher areal power and energy.

In summary, we have developed an effective and general strategy to create a new class of 1D flexible hierarchically structured “sheath–core” metallic scaffold by assembling bicontinuous porous Ni framework onto various solid metal wires via template‐directed assembly and electrodeposition. The resultant metallic scaffold provides a new and robust platform to evaluate a wide range of active materials including metal oxides and conductive polymers in unconventional 1D fiber device configuration. As compared to previous 1D carbonaceous/metallic electrode architectures, our well‐engineered 1D flexible scaffolds are endowed with interconnected open‐pore metallic network with macroporous feature, which not only guarantees a desirably high electroactive material loading (1 mg cm^−2^) in such tiny fiber format, but also renders rapid and efficient electronic and ionic transport “highways,” thereby boosting the energy storage capability of pseudocapacitive materials (e.g., MnO_2_ nanostructures) to their theoretical limits in 1D fiber electrodes. As expected, the optimized 1D metallic scaffold supported‐MnO_2_ hybrid fiber electrode yields ultrahigh specific areal capacitance of 1303.61 mF cm^−2^ (≈1278 F g^−1^ based on MnO_2_, approaching the theoretical value of ≈1370 F g^−1^) in liquid KOH electrolyte and 847.22 mF cm^−2^ in PVA/KOH gel electrolyte, which is 2–350 times of previously reported fiber electrodes. The assembled solid‐state fiber micro‐pseudocapacitor simultaneously achieves remarkable areal energy and power densities of 18.83 µWh cm^−2^ and 16.33 mW cm^−2^ (75.32 µWh cm^−2^ and 65.32 mW cm^−2^ based on single electrode), greatly exceeding the values of the existing symmetric fiber SCs (0.17–9.2 µWh cm^−2^ and 0.1–4.2 mW cm^−2^) and some macroscopic pseudocapacitors, together with very long cycle life and good rate capability. Impressively, the maximum areal energy output even competes favorably with some state‐of‐the‐art microbatteries, while the maximum power density is one to three orders of magnitude higher than those of microbatteries. Furthermore, our fiber device is flexible and highly durable in bending tests. In principle, our proposed strategy can be regarded as a general approach and applicable to other pseudocapacitive materials to extract their huge potential for advanced fiber micro‐pseudocapacitors. Our versatile strategy can also produce textile electrodes based on metal gauzes for textile energy storage. Thus, the present studies open new opportunities for exploiting high performance flexible fiber/textile electrodes for next‐generation of energy storage devices, and our developed fiber pseudocapacitors with combined high power and energy densities have great promise in flexible/portable electronics, functional textiles, sensors, and beyond.

## Supporting information

Supplementary
