# Supplementary material for: A General Electrode Design Strategy for Flexible Fiber Micro‐Pseudocapacitors Combining Ultrahigh Energy and Power Delivery
Source: Adv Sci (Weinh). 2017 Mar 3;4(8):1700003. doi: 10.1002/advs.201700003 (PMC5566233; doi:10.1002/advs.201700003)
Supplement: Supplementary file 1 — Supplementary [file ADVS-4-na-s001.pdf]

Copyright WILEY-VCH Verlag GmbH & Co. KGaA, 69469 Weinheim, Germany, 2017.

ADVANCED SCIENCE Open Access

Supporting Information

for *Adv. Sci.*, DOI: 10.1002/advs.201700003

A General Electrode Design Strategy for Flexible Fiber Micro-Pseudocapacitors Combining Ultrahigh Energy and Power Delivery

Ping Li, Jing Li, Zhe Zhao, Zhengsong Fang, Meijia Yang, Zhongke Yuan, You Zhang, Qiang Zhang, Wei Hong, Xudong Chen,* and Dingshan Yu**

Copyright WILEY-VCH Verlag GmbH & Co. KGaA, 69469 Weinheim, Germany, 2016.

Supporting Information

A General Electrode Design Strategy for Flexible Fiber Micro-Pseudocapacitors Combining Ultrahigh Energy and Power Delivery

Ping Li, + Jing Li, + Zhe Zhao, Zhengsong Fang, Meijia Yang, Zhongke Yuan, You Zhang, Qiang Zhang, Wei Hong, Xudong Chen,* and Dingshan Yu**

Fabrication of 1D porous nickel scaffold. Nickel wires with diameter of 0.1 mm (Alfa Aesar) were rinsed with ethanol prior to use. Polystyrene (PS) microspheres with the diameter of 2 μm were dispersed in water with a concentration of 3 mg/mL to form stable suspensions, respectively. A nickel plates (1×4 cm) and the nickel wires were employed as counter (cathode) and working (anode) electrodes, respectively, with a distance of ~2.0 cm in the dispersion. The colloidal templates were formed on the nickel wire through electrophoretic deposition with a voltage of 8 V for 6 minutes. Afterwards, the templates on the nickel wire were sintered at 112 °C for 1 h to increase the interconnection size between the PS microspheres. After immersed in ethanol to ensure the full infiltration, nickel was electrodeposited onto the colloidal template to form macroporous nickel framework through templates at a constant current density of 2 mA cm^{-2} with a nickel plate as a counter electrode. The PS was removed by immersing the plated fibers in chloroform for 2 hours with oscillation followed by 10 minutes oxygen plasma etching for removing the residual polymer.

MnO_2 electrodeposition on 1D porous Ni scaffold. Anodic pulse (6 mA cm^{-2}, 0.225 s on, 4 s off, 17 min) deposition were applied to the fiber-like nickel scaffold in the mixed solution of 0.5 M $MnSO_4$ and 0.5 M CH_3COONa at 35 °C with a platinum counter electrode. The fiber based nickel scaffold with 1500 (6 min), 3000 (12 min), 5000 (19 min) and 6000 (23 min) MnO_2 deposited cycles were prepared to achieve various MnO_2 loading. The mass loading was calculated via charge flow throughout the electrodeposition process.

Electrochemical Characterization of Individual Fiber Electrode. A three-electrode cell setup with a CHI 760E potentiostat, consisting of a single fiber, a large-area Pt foil and a

saturated calomel (SCE) electrode as the working electrode, a reference, and a counter electrode, respectively, was used for capacitance measurements in 6 M KOH electrolyte. Electrochemical impedance spectra (EIS) were measured at frequencies ranging from 0.01 to 100 000 0 Hz with a potential amplitude of 5 mV. The specific capacitance ($C_{sp,x}$) of the fibers in a three-electrode cell was calculated using the charge integrated from charge/discharge (GCD) and CV curves individually according to the formulas:

$$C_{sp,\,x} = \frac{It}{UX} \quad (1)$$

$$C_{sp,\,x} = \frac{Q}{2UX} = \frac{1}{2UvX}\int_{U-}^{U+} i(U)dU \quad (2)$$

where I is the discharge current during GCD, t is the discharge time during GCD, U ($U=U_+ - U_-$) represents the scanned potential window of 0.8 V used in the three-electrode cell in this study, Q is the total voltammetric charge obtained by integrating the positive and negative sweeps (i(U)is the current) of a CV curve, and v is the scan rate of the CV curve. Where X could be surface area (A) and effective length (L) for area-specific capacitance ($C_{sp,A}$), length-specific capacitance ($C_{sp,L}$), respectively. The surface area (S) of the fiber was determined according to the formulas:[3]

$$S = \pi DL \quad (3)$$

where D is the diameter of the fiber.

Fabrication and Electrochemical Characterization of All-Solid-State Fiber Supercapacitors. To prepare the polymeric gel electrolyte (PVA/KOH), KOH (3 g) was added to de-ionized water (60 mL), followed by the addition of a PVA powder (6 g). The above mixture was heated to 85 °C under vigorous stirring until it became clear. Two identical dry fibers with the same length were immersed in the PVA/KOH electrolyte solution for 5 min. Thereafter, the electrolyte wetted fibers were placed on a PET film in parallel and dried until the PVA/KOH gel solidification under ambient conditions. The performance of the assembled fiber supercapacitors was evaluated by CV and galvanostatic charge/discharge (GCD) in a two-electrode configuration using the potentiostat (CHI 760E). The capacitance of the single electrode ($C_{s,\ electrode}$) in a two-electrode cell was calculated from GCD and CV curves individually according to the formulas:[3]

$$C_{s,\,electrode} = \frac{2It}{U} \tag{5}$$

$$C_{s,\,electrode} = \frac{1}{Uv}\int_{U_-}^{U_+} i(U)dU \tag{6}$$

The capacitance of the device ($C_{cell,\,x}$) is equal to the half of $C_{s,\,electrode}$. The device areal and length of the supercapacitors were calculated according to the equations:

$$C_{cell,\,x} = \frac{C_{cell,\,device}}{X_{cell}} \tag{7}$$

$$X_{cell} = 2X \tag{8}$$

where X_{cell} could be device area (A_{cell}), effective length (L_{cell}) of the fiber-supercapacitors, respectively.

The areal energy density of the supercapacitors ($E_{cell,s}$) was obtained from the equation:

$$E_{cell,\,s} = \frac{C_{cell,\,s} \times \Delta E^2}{2 \times 3600} \tag{9}$$

where ΔE is the operating voltage window in volts.

The areal power density was calculated from the GCD curves at different charge/discharge current densities using the equation:

$$P_{cell,\,s} = \frac{E_{cell,\,s} \times 3600}{t_{discharge}} \tag{10}$$

where $t_{discharge}$ is the discharge time.

Characterization. The surface structure and morphology for all the samples were characterized using a Hitachi S-4800 scanning electron microscope (SEM) equipped with energy dispersive X-ray (EDX) element mapping. Transmission electron microscopic (TEM) images were taken on JEOL JEM-2100F field-emission microscope. The X-ray photoelectron spectroscopy (XPS, ESCALAB 250) was used to analyze the chemical state of the deposited MnO_2. The crystalline structure of the deposited MnO_2 was examined on an X-ray diffraction instrument (Bruker AXS D8).

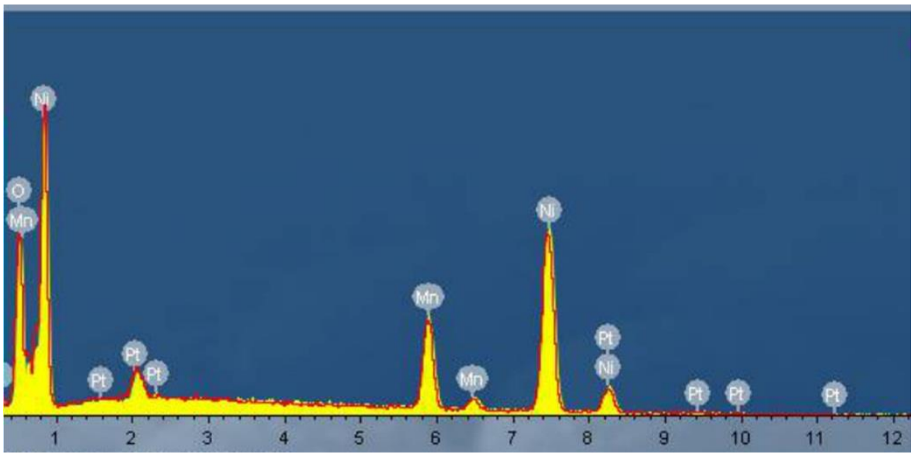

Figure S1. Energy-dispersive X-ray (EDX) spectrum of porous Ni framework sheathed metal Ni wire with a thin MnO_2 plating layer.

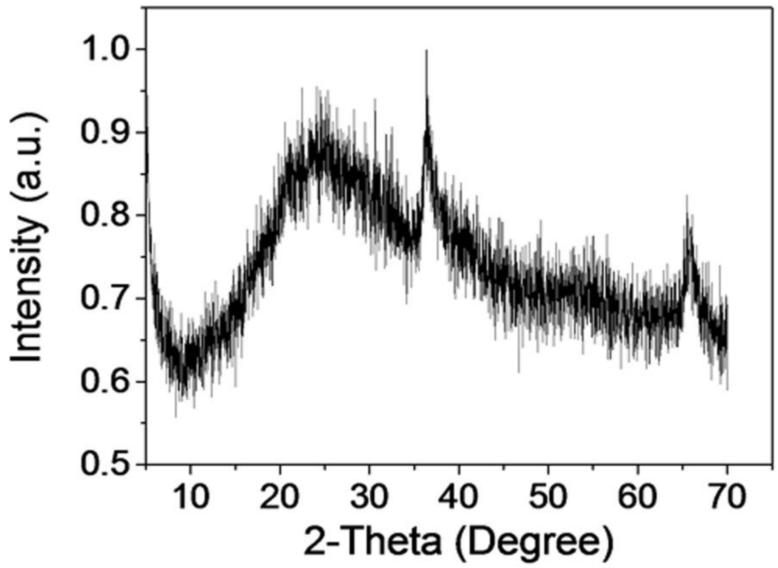

Figure S2. XRD pattern of the deposited MnO_2. The peaks located at 24°, 37° and 66° can be indexed to birnessite-type MnO_2 (JCPDS 42-1317).[1] Lack of sharp peaks and broadening of peaks indicates the poor crystallization and approximately amorphous nature of the as-deposited MnO_2 nanoparticles.

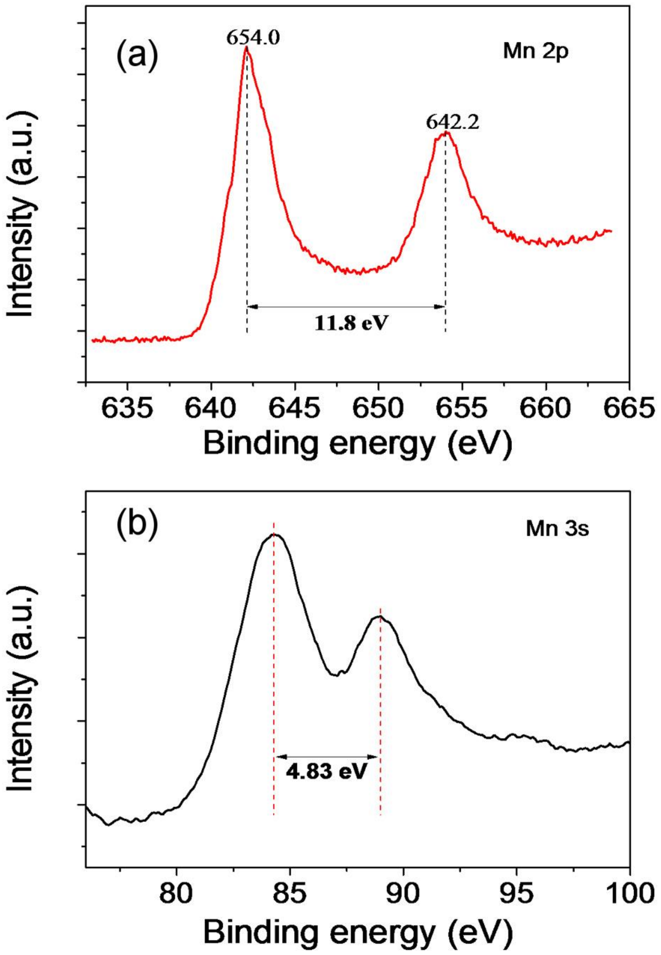

Figure S3. High-resolution XPS spectra of the MnO_2: (a) Mn 2p and (b) Mn 3s.

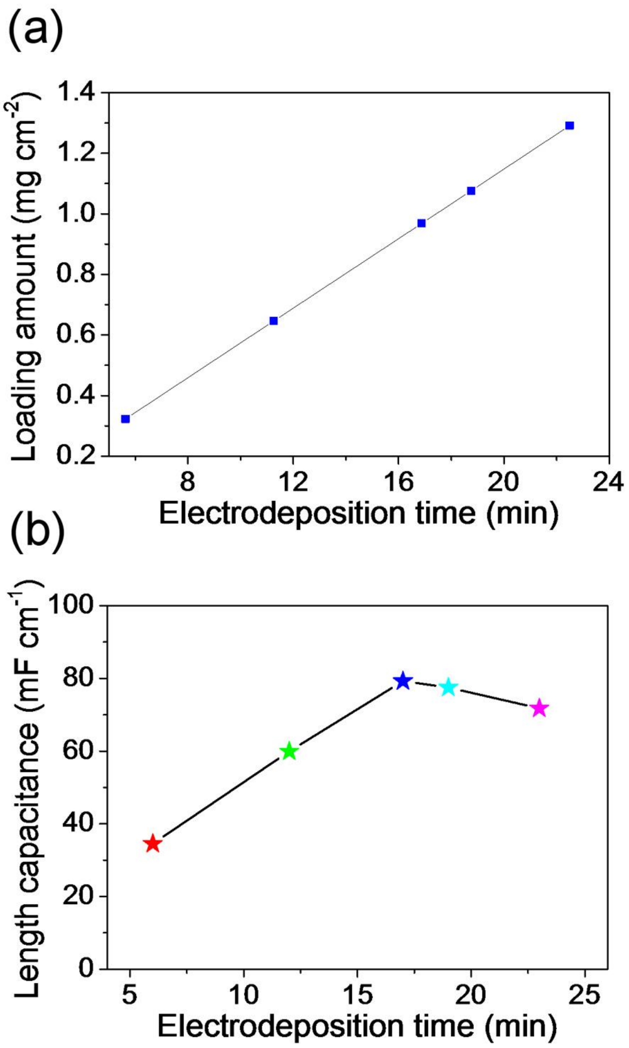

Figure S4. The plots of (a) the areal loading mass of MnO_2 *versus* the electrodeposition time and (b) the length capacitance *versus* the electrodeposition time. Length capacitances are calculated from the CV result at 5 mV/s in 6 M KOH aqueous electrolyte measured in a three-electrode cell.

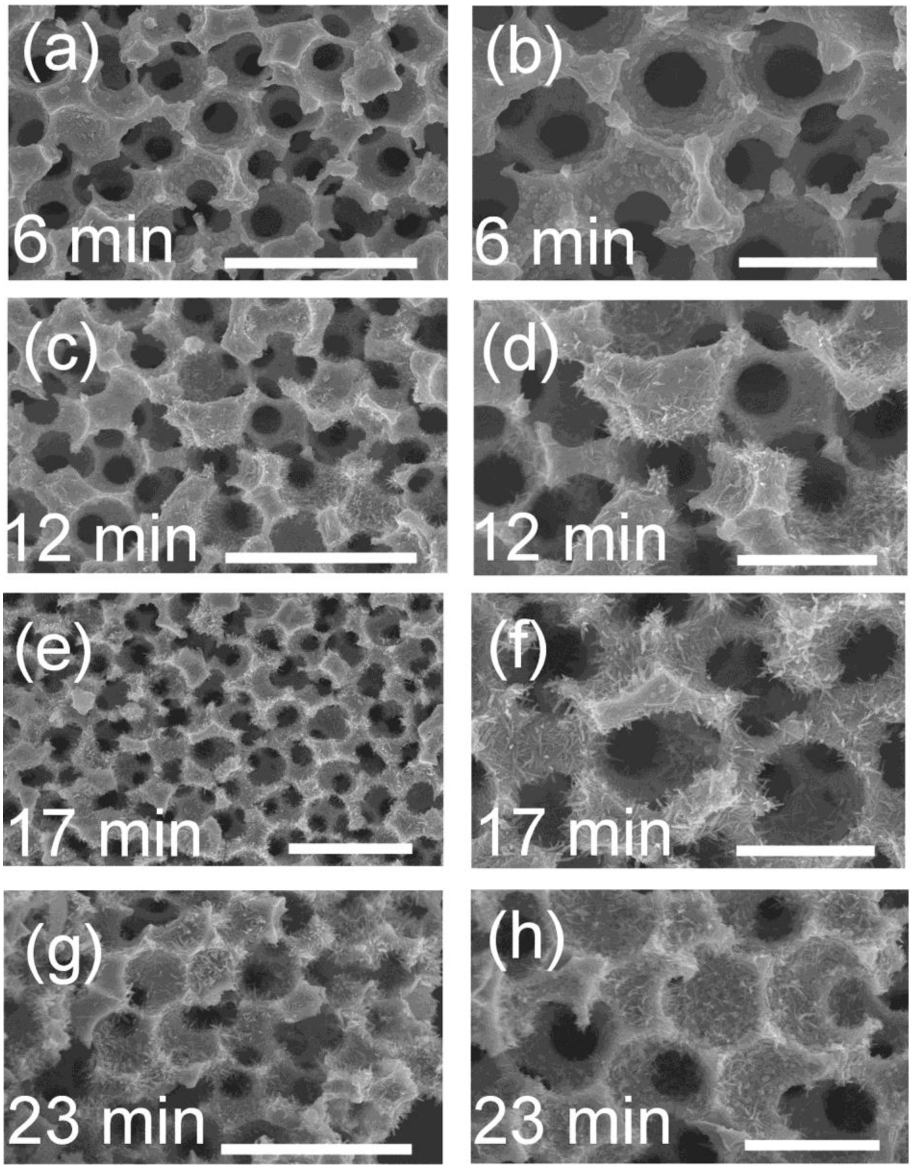

Figure S5. (a) Low- and (b) high-magnification SEM images of the MnO_2/porous Ni with 6 min deposited time. (c) Low- and (d) high-magnification SEM images of the MnO_2/porous Ni with 12 min deposited time. (e) Low- and (f) high-magnification SEM images of the MnO_2/porous Ni with 17 min deposited time. (g) Low- and (h) high-magnification SEM of the MnO_2/porous Ni with 23 min deposited time. Scale bars: 5 μm for (a), (c), (e) and (g), respectively, and 2 μm for (b), (d), (f) and (h), respectively.

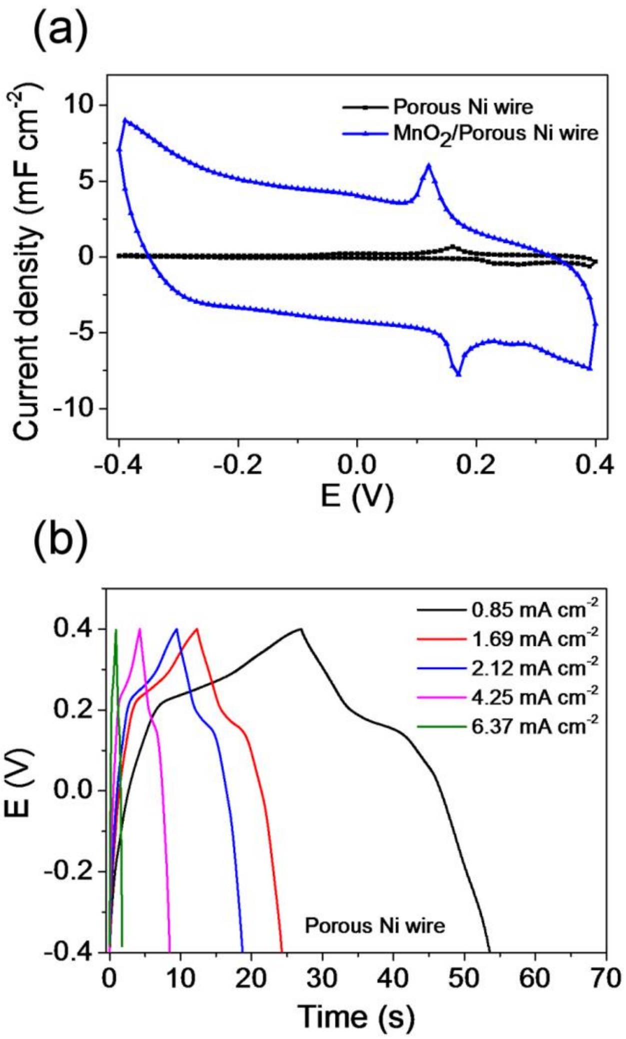

Figure S6. (a) CV curves of porous Ni wire and MnO_2/porous Ni wire measured at 5 mV/s in 6 M KOH electrolyte. (b) Galvanostatic charge/discharge curves of porous Ni wire without MnO_2 at various current densities from 0.85 to 6.37 mA/cm^2.

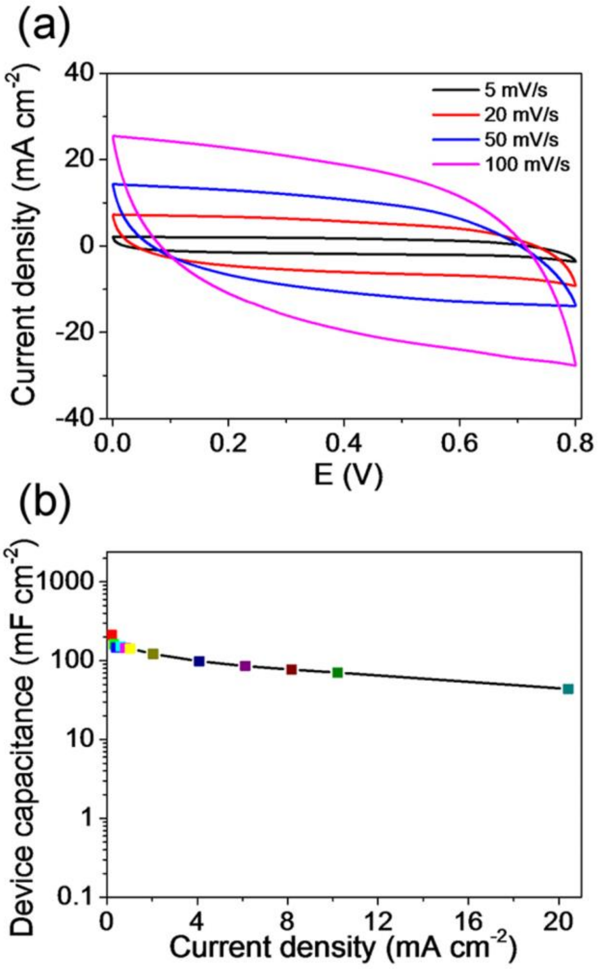

Figure S7. (a) CV curves of the optimized fiber supercapacitor based on the MnO_2/porous Ni wire at various scan rates from 5 to 100 mV/s. (b) Areal device capacitance of the optimized fiber supercapacitors as a functon of applied current densities.

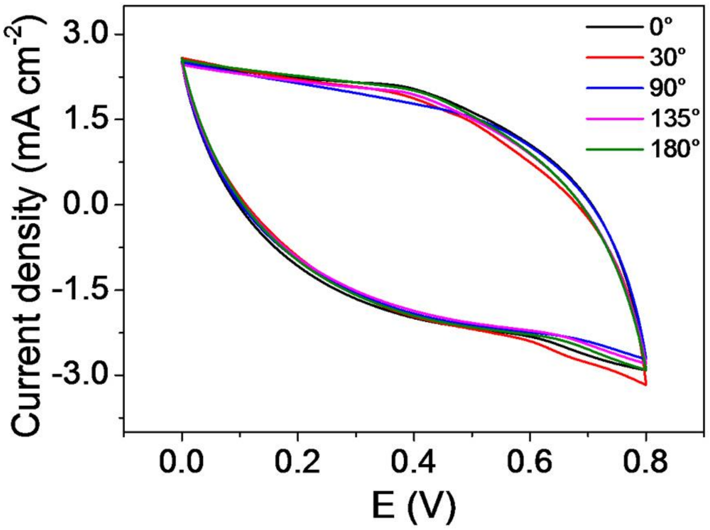

Figure S8. CV curves of the optimized fiber supercapacitor based on the MnO_2/porous Ni wire at different bending angles.

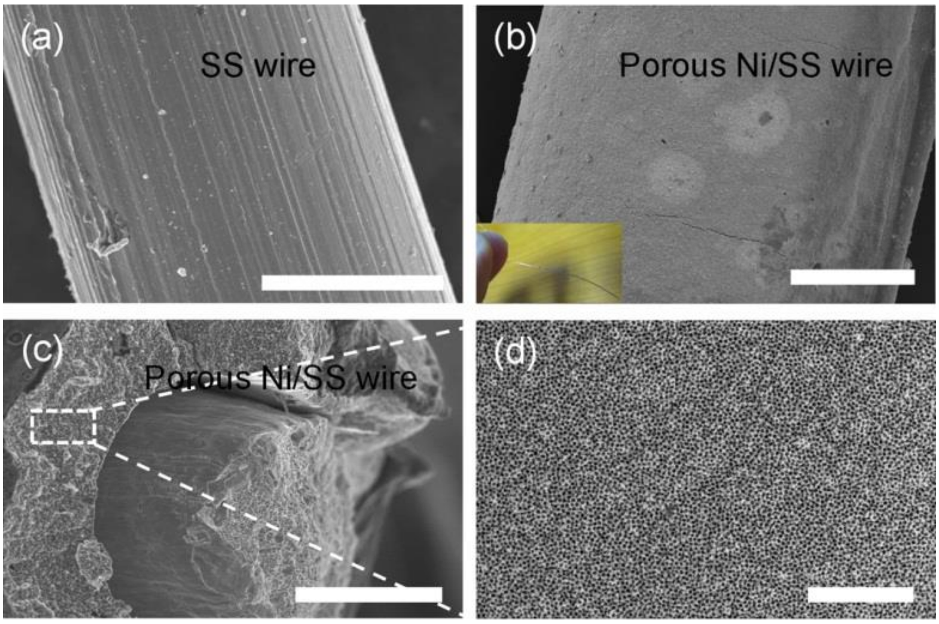

Figure S9. (a) SEM image of the bare stainless steel (SS) wire. (b) SEM image of bicontinuous porous Ni framework sheathed metal SS wire. The inset is digital photograph of porous Ni framework sheathed metal SS wire. (c) SEM image of the fractured surface and side-section of the porous Ni framework sheathed metal SS wire. (d) SEM image of the square area highlighted in (c). Scale bars: 60 μm (a), 100 μm (b), 50 μm (c) and 10 μm (d).

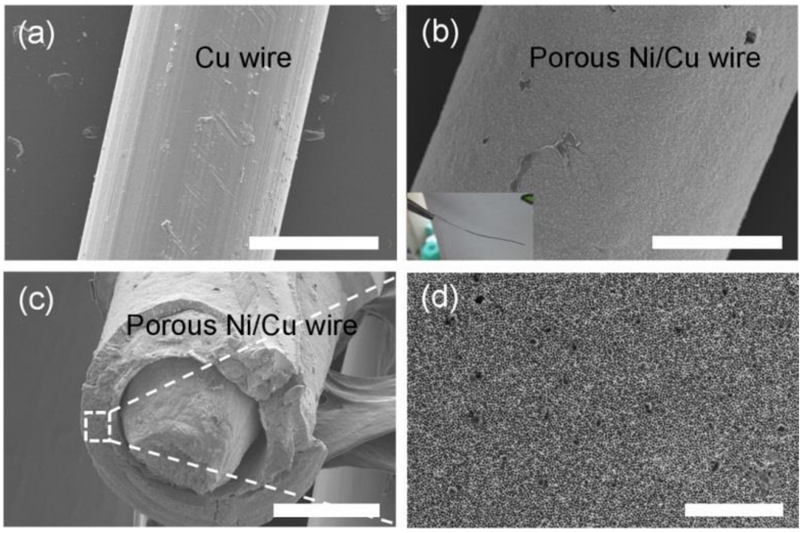

Figure S10. (a) SEM image of the bare Cu wire. (b) SEM image of bicontinuous porous Ni framework sheathed metal Cu wire. The inset is digital photograph of porous Ni framework sheathed metal Cu wire. (c) SEM image of the fractured surface of the porous Ni framework sheathed metal Cu wire . (d) SEM image of the square area highlighted in c. Scale bars: 100 μm (a), 50 μm (b), 100 μm (c) and 10 μm (d).

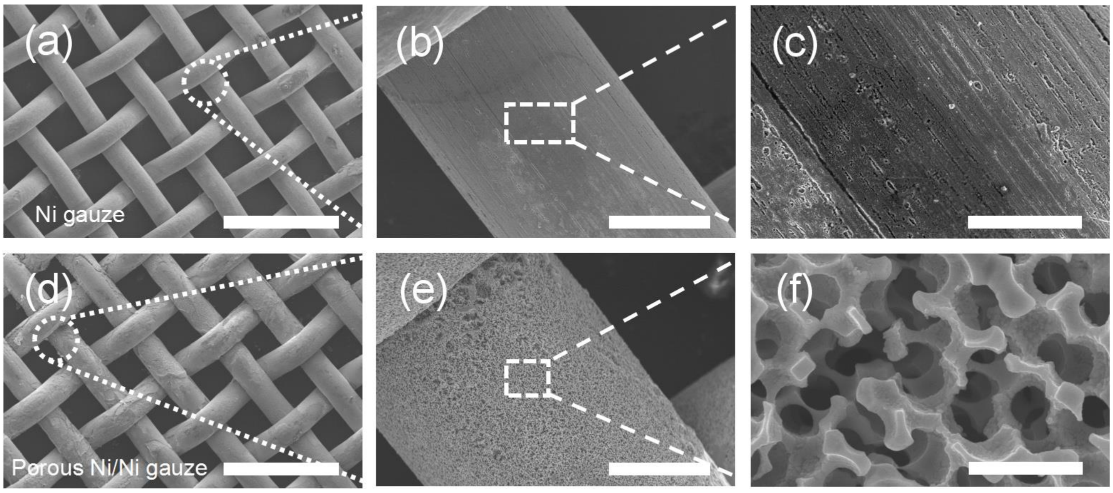

Figure S11. (a) SEM image of the bare Ni gauze. (b) High magnification SEM image of the circular area highlighted in (a). (c) High magnification SEM image of the square area highlighted in (b). (d) SEM image of the Ni gauze sheathed with porous Ni framework. (e) High magnification SEM image of the circular area highlighted in (d). (f) High magnification SEM image of the square area highlighted in (e). Scale bars: 60 μm (a) and (d), 50 μm (b) and (e), 20 μm (c) and 2 μm (f).

Table S1. The comparison of electrochemical performance of selective recently-reported yarn- and fiber-based supercapacitors.

**Electrode materials**	**Electrolyte materias**	**$C_{L,single}$ (mF cm^{-1})**	**$C_{s,single}$ (mF cm^{-2})**	**$C_{s,device}$ (mF cm^{-2})**	**E_A (μWh cm^{-2})**	**P_A (mW cm^{-2})**	**Ref.**
MnO_2/Porous Ni fiber	PVA/KOH	79.8	847.2	211.8	18.83	16.33	**This Work**
PEDOT/MWCNT Biscrolled yarn	PVA/H_2SO_4		73				*Nat. Commun*. **2013**, *4*, 1970.
SWCNT/rGO fibre	PVA/H_3PO_4			116.3			*Nat. Nanotechnol*. **2014**, *9*, 555.
rGO-Ni yarn	PVA/H_3PO_4	13.0	72.1		1.60	2.42	*Adv. Mater.* **2016**, *28*, 98.
Hollow rGO/PEDOT:PSS fiber	PVA/H_3PO_4	8.1	304.5		6.8	0.166	*Adv. Mater*. **2016**, *28*, 3646.
PPy@MnO_2@rGO-deposited conductive yarns	PVA/H_3PO_4	31	411		9.2	1.33	*ACS Nano* **2015**, *9*, 4766.
MnO_2/CNT/Nylon fiber	PVA/LiCl			40.9	2.6	0.0669	*Sci. Rep*. **2015**, *5*, 9387.
PPy@CNTs@Urethane elastic fiber core spun yarn	PVA/H_3PO_4			67	6.13	0.133	*Nano Energy* **2016**, *27*, 230.
CNT@Co_3O_4 yarn	PVA/H_2SO_4		52.6		1.10	0.01	*Small* **2015**, *11*, 854.
MnO_2/ZnO nanowires/ Au–PMMA wire	PVA/H_3PO_4	0.2	2.4		0.03(single)	0.014(single)	*Angew. Chem. Int. Ed*. **2011**, *50*, 1683.
rGO/SWCNT@ Carboxymethyl cellulose	PVA/H_3PO_4	5.3	177		3.84	0.19	*Nat. Commun*. **2014**, *5*, 3754.
SWCNT/Activated carbon yarn	PVA/H_2SO_4		37.1		0.8		*Adv. Mater.* **2014**, *26*, 4100.
CNT fiber	PVA/H_2SO_4		4.28		0.226	0.493	*Adv. Energy Mater.* **2014**, *4*, 1300759.
MnO_2/rGO fiber	PVA/H_2SO_4	0.143	9.6				*J. Power Sources* **2014**, *247*, 32.

PANI nanowire array/CNT yarn	PVA/H_3PO_4		38		0.84		*Adv. Mater*. **2013**, *25*, 1494.
MWCNT/Carbon fiber	PVA/H_2SO_4			71.1	9.8	0.189	*ACS Nano* **2013**, *7*, 5940.
Mesoporous carbon/ CNT yarn	PVA/H_3PO_4	1.91	39.7		0.86		*Adv. Mater.* **2013**, *25*, 5965.
Bi_2O_3 nanotubes -loaded graphene fibers	PVA/H_3PO_4		69.3	17.3	1.5		*Nanoscale* **2014**, *6*, 8595.
PANI/CNT	PVA/H_2SO_4			6.2	0.57	0.145	*Small* **2014**, *10*, 3187.
CNT/Carbon fiber	PVA/H_3PO_4			1.7	0.17	0.1	*Adv. Mater*. **2013**, *25*, 2326.
PANI-Stainless steel wire	PVA/H_2SO_4			19	0.95	4.2	*Energy Environ. Sci*. **2013**, *6*, 805.
Nanoporous gold @MnO_2//CNT/Carbon	PVA/LiCl			12	5.4	2.53	*Nano Res*. **2015**, *8*, 1148.
MnO_2/CNT-coiled fiber	PVA/LiCl	2.72		61.25	8.5		*Adv.Energy Mater*. **2016**, *6*, 1502119.
rGO/Au wire	PVA/H_3PO_4	0.1	6.49				*Chem. Commun*. **2013**, *49*, 291.
rGO/Ni-cotton yarn	PVA/LiCl	110					*Nat. Commun*. **2015**, *6*, 7260.
MnO_2/CNT fiber//CNT fiber	PVA/KOH	0.13-0.15		13.89- 15.85			*ACS Nano* **2015**, *9*, 6088.
Multi-channeled CNF yarns	PVA/H_3PO_4			91			*Energy Storage Materials*. **2016**, *5*, 43.

References for Supporting Information

[1] Z. J. Fan, J. Yan, T. Wei, L. J. Zhi, G. Q. Ning, T. Y. Li, F. Wei, *Adv. Funct. Mater.* **2011**, *21*, 2366
[2] J. A. Lee, M. K. Shin, S. H. Kim, H. U. Cho, G. M. Spinks, G. G. Wallace, M. D. Lima, X. Lepro´, M. E. Kozlov, R. H. Baughman, S. J. Kim, *Nat. Commun*. **2013**, *4*, 1970.
[3] D. S. Yu, K. L. Goh, H. Wang, L. Wei, W. C. Jiang, Q. Zhang, L. M. Dai, Y. Chen, *Nat. Nanotechnol*. **2014**, *9*, 555.
[4] X. Pu, L. X. M. M. Liu, C. Y. Jiang, C. H. Du, Z. F. Zhao, W. G. Hu, Z. L. Wang, *Adv. Mater.* **2016**, *28*, 98.
[5] G. X. Qu, J. L. Cheng, X. D. Li, D. M. Yuan, P. N. Chen, X. L. Chen, B. Wang, H. S. Peng, *Adv. Mater*. **2016**, *28*, 3646.
[6] Y. Huang, H. Hu, Y. Huang, M. S. Zhu, W. J. Meng, C. Liu, Z. X. Pei, C. L. Hao, Z. K. Wang, C. Y. Zhi, *ACS Nano* **2015**, *9*, 4766.
[7] C. Choi, S. H. Kim, H. J. Sim, J. A. Lee, A. Y. Choi, Y. T. Kim, X. Lepro´, G. M. Spinks, R. H. Baughman, S. J. Kim, *Sci. Rep.* **2015**, *5*, 9387.
[8] J. F. Sun, Y. Huang, C. X. Fu, Z. Y. Wang, Y. Huang, M. S. Zhu, C. Y. Zhi, H. Hu, *Nano Energy* **2016**, *27*, 230.
[9] F. H. Su, X. M. Lv, M. H. Miao, *Small* **2015**, *11*, 854.
[10] J. Bae, M. K. Song, Y. J. Park, J. M. Kim, M. L. Liu, Z. L. Wang, *Angew. Chem. Int. Ed.* **2011**, *50*, 1683.
[11] L. Kou, T. Q. Huang, B. N. Zheng, Y. Han, X. L. Zhao, K. Gopalsamy, H. Sun, C. Gao, *Nat. Commun*. **2014**, *5*, 3754.
[12] Q. H. Meng, H. P. Wu, Y. N. Meng, K. Xie, Z. X. Wei, Z. X. Guo, *Adv. Mater.* **2014**, *26*, 4100.
[13] P. Xu, T. L. Gu, Z. Y. Cao, B. Q. Wei, J. Y. Yu, F. X. Li, J.-H. Byun, W. B. Lu, Q. W. Li, T.-W. Chou, *Adv. Energy Mater.* **2014**, *4*, 1300759.
[14] Q. Chen, Y. N. Meng, C. G. Hu, Y. Zhao, H. B. Shao, N. Chen, L.T. Qu, *J. Power Sources* **2014**, *247*, 32.
[15] K. Wang, Q. H. Meng, Y. J. Zhang, Z.X. Wei, M. H. Miao, *Adv. Mater*. **2013**, *25*, 1494.
[16] V. T. Le, H. Kim, A. Ghosh, J. Kim, J. Chang, Q. A. Vu, D. T. Pham, J.-H. Lee, S.-W. Kim, Y. H. Lee, *ACS Nano* **2013**, *7*, 5940.
[17] J. Ren, W. Y. Bai, G. Z. Guan, Y. Zhang, H. S. Peng, *Adv. Mater.* **2013**, *25*, 5965.
[18] K. Gopalsamy, Z. Xu, B. N. Zheng, T. Q. Huang, L. Kou, X. L. Zhao, C. Gao, *Nanoscale* **2014**, *6*, 8595.
[19] Q. H. Meng, K. Wang, W. Guo, J. Fang, Z. X. Wei, X. L. She, *Small* **2014**, *10*, 3187.
[20] Y. Meng, Y. Zhao, C. G. Hu, H. H. Cheng, Y. Hu, Z. P. Zhang, G. Q. Shi, L. T. Qu, *Adv. Mater*. **2013**, *25*, 2326.
[21] Y. P. Fu, H. W. Wu, S. Y. Ye, X. Cai, X. Yu, S. C. Hou, H. Kafafy, D. C. Zou, *Energy Environ. Sci*. **2013**, *6*, 805.
[22] H. H. Xu, X. L. Hu, Y. M. Sun, H. L. Yang, X. X. Liu, Y. H. Huang, *Nano Res*. **2015**, *8*, 1148.
[23] C. Choi, H. J. Sim, G. M. Spinks , X. Lepró, R. H. Baughman, S. Kim, *Adv. Energy Mater*. **2016**, *6*, 1502119.
[24] Y. R. Li, K. X. Sheng, W. J. Yuan, G. Q. Shi, *Chem. Commun*. **2013**, *49*, 291.
[25] L. B. Liu, Y. Yu, C. Yan, K. Li, Z. J. Zheng, *Nat. Commun*. **2015**, *6*, 7260.
[26] P. Xu, B. Q. Wei, Z. Y. Cao, J. Zheng, K. Gong, F. X. Li, J. Y. Yu, Q. W. Li, W. B. Lu, J.-H. Byun, B.-S. Kim, Y. S. Yan, T.-W. Chou, *ACS Nano* **2015**, *9*, 6088.
[27] L. Shi, X. L. Li, Y. Y. Jia, D. B. Kong, H. Y. He, M. Wagner, K. Müllen, L. J. Zhi, *Energy Storage Materials*. **2016**, *5*, 43.
